# Entropy and Variational Formulation of Relativistic Fluid Dynamics

**DOI:** 10.3390/e27121260

**Published:** 2025-12-16

**Authors:** Asher Yahalom

**Affiliations:** 1 Department of Electrical & Electronic Engineering, Faculty of Engineering, Ariel University, Ariel 40700, Israel; asya@ariel.ac.il; Tel.: +972-54-7740294; 2 Center for Astrophysics, Geophysics, and Space Sciences (AGASS), Ariel University, Ariel 40700, Israel; 3 FEL User Center, Ariel University, Ariel 40700, Israel

**Keywords:** non-barotropic flows, variational analysis, relativistic flows

## Abstract

In this work, the earlier variational analysis of classical non-barotropic flows is extended to the special relativistic non-barotropic case. Specifically, we present a new Eulerian variational formulation for relativistic non-barotropic flows, based on six functions. This allows the canonical derivation of the energy–momentum tensor.

## 1. Introduction

A detailed overview of the variational formulation of non-relativistic fluid dynamics, along with their profound interrelations, can be found in [1,2]; here, we provide a short description of the history of the subject.

Variational principles for barotropic fluid dynamics are described in the literature. A four-function variational principle for a Eulerian barotropic fluid was described by Clebsch [3,4] and much later by Davidov [5], whose main purpose was to quantize fluid dynamics. The work was written in Russian and was largely unknown in the West. Lagrangian fluid dynamics (which takes a different approach compared to Eulerian fluid dynamics) was given a variational description by Eckart [6]. Ignoring both the work of Clebsch (written in German) and the work of Davidov (written in Russian), initial attempts in the English literature to formulate Eulerian fluid dynamics using a variational principle were given by Herivel [7], Serrin [8] and Lin [9]. However, the variational principles developed by the above authors were cumbersome, relying on quite a few “Lagrange multipliers” and auxiliary “potentials”. The total number of independent functions in the above formulations ranges from eleven to seven, which is much larger than the four functions required for the Eulerian and continuity equations of a barotropic flow. Thus, these methods did not have practical use. Seliger and Whitham [10] have reintroduced the variational formalism of Clebsch, depending on only four variables, for a barotropic flow. Bretherton [11], has noted that for knotted vortex lines the Clebsch form must include a non single valued function. Lynden-Bell and Katz [12] have described a variational principle in terms of two functions: the load λ and density ρ. However, their formalism contains an implicit definition for the velocity $\vec{v}$ such that one is required to solve a partial differential equation in order to obtain both $\vec{v}$ in terms of ρ and λ as well as its variations. Much the same criticism holds for the general variational formulations for non-barotropic flows [13]. Yahalom and Lynden-Bell [14] overcame the implicit definition limitation by adding an additional single variational variable. This formalism allows arbitrary variations (not constrained) and the definition of $\vec{v}$ is explicit.

The pioneering studies by Clebsch [3,4] and the subsequent related works all consider non-relativistic fluids, where the flow velocity is much smaller than the speed of light in a vacuum, *c*. This is naturally understandable, since Clebsch’s research predates Einstein’s theory of special relativity by forty-eight years. In practice, relativistic fluid flows are rarely observed under terrestrial conditions.

The conventional treatment of ideal relativistic flows relies on the energy–momentum tensor [15,16,17]. However, modern relativistic hydrodynamics of non-ideal flows is not formulated solely in terms of the energy–momentum tensor, except in the perfect fluid case. In general, one works with a set of constitutive relations and hydrodynamic equations, as summarized, for instance, in Section 2.1 of [18]. In such cases, the energy–momentum tensor has to be defined consistently with the Bianchi identities and the choice of the fundamental dynamical fields. It must also be consistent with the second law of thermodynamics in its local form, as well as any possible extra conservation law that physics enforces. We also note the multifluid approach suggested in [19].

In the ideal case, it seems possible to obtain a canonical energy–momentum tensor if one can properly define a Lagrangian density for such a system [20]. The Lagrangian density of an ideal fluid in terms of a Lagrangian fluid picture in a general relativistic context was described in [21,22] (Section 3.10 and references therein). An interesting approach to obtaining the Lagrangian is described in [23] (Chapter 6). The Lagrangian density of an ideal fluid in terms of a Eulerian fluid in a general relativistic context was described in [24]. In a more recent work [25], Clebsch’s formulation was extended to the special relativistic regime, thereby deriving a Lagrangian density in the Eulerian context for relativistic fluids, from which the corresponding energy–momentum tensor for high-velocity flows can be obtained in a consistent manner. Moreover, a link between relativistic fluid dynamics and the Dirac theory of the electron, established through the concept of Fisher information, was discussed in [26]. Nonetheless, ref. [25] does not address non-barotropic Eulerian flows, focusing instead on connections to quantum mechanics—a limitation that the present work seeks to resolve. The current work is thus less general than [24] as it addresses only a special relativistic case and not a general relativistic case; however, it assumes the possibility that the fluid may be charged, and, in this sense, it is more general than [24].

The formulation of the variational principle for a relativistic charged classical particle interacting through a vector potential, as well as for a system of such particles, has been presented in [25] and will not be repeated here. Nonetheless, we will adopt the notation introduced in [25] whenever required.

We begin with Eckart’s [6] Lagrangian variational principle, extended to describe a relativistic charged fluid. This is followed by the presentation of the general theory of the canonical energy–momentum tensor, from which we derive the tensor corresponding to the Eckart Lagrangian. Next, we introduce a Eulerian–Clebsch variational formulation for a non-barotropic relativistic charged fluid and obtain its associated energy–momentum tensor. Finally, we examine the cosmological implications of this tensor.

## 2. The Lagrangian Description of a Relativistic Charged Fluid

### 2.1. Action and Lagrangian

The dynamics of a flow are determined by its composition and the forces acting upon it. The fluid is considered to be composed of “fluid elements” [6,11]. Each fluid element may be viewed as a point-like entity with infinitesimal mass $dM_{\vec{\alpha }}$, charge $dQ_{\vec{\alpha }}$, position four-vector $x_{\vec{\alpha }\nu }\left(\tau_{\vec{\alpha }}\right)$, and four-velocity $u_{\vec{\alpha }\nu }\left(\tau_{\vec{\alpha }}\right)\equiv \frac{dx_{\vec{\alpha }\nu }\left(\tau_{\vec{\alpha }}\right)}{d\tau_{\vec{\alpha }}}$. Here, the continuous label $\vec{\alpha }$ replaces the discrete index *n* used in the particle-based description (see Section 2 of [25]). However, a fluid element is not a true point particle, as it possesses an infinitesimal volume $dV_{\vec{\alpha }}$, entropy $dS_{\vec{\alpha }}$, and internal energy $dE_{\mathrm{in},\vec{\alpha }}$. The action for each fluid element, following Equation (1) of [25], takes the form:

(1) $$\begin{array}{c} dA_{\vec{\alpha }}=-dM_{\vec{\alpha }}c\int d\tau_{\vec{\alpha }}-dQ_{\vec{\alpha }}\int A^{\mu }\left(x_{\vec{\alpha }}^{\nu }\right)dx_{\mu \vec{\alpha }}+dA_{in\;\vec{\alpha }},\\ dA_{in\;\vec{\alpha }}\equiv -\int dE_{in\;\vec{\alpha }}dt. \end{array}$$

The Lagrangian corresponding to each “fluid element” can be obtained from the above expression in the following way (the symbol ≃ represents here the low velocity (classical) approximation):

(2) $$\begin{array}{ccc} dA_{\vec{\alpha }} & = & \int_{t1}^{t2}dL_{\vec{\alpha }}dt,\;dL_{\vec{\alpha }}\equiv dL_{k\vec{\alpha }}+dL_{i\vec{\alpha }}-dE_{in\;\vec{\alpha }}\\ dL_{k\vec{\alpha }} & \equiv & -\frac{dM_{\vec{\alpha }}c^{2}}{\gamma_{\vec{\alpha }}}≃\frac{1}{2}dM_{\vec{\alpha }}\;v_{\vec{\alpha }}{\left(t\right)}^{2}-dM_{\vec{\alpha }}c^{2}\\ dL_{i\vec{\alpha }} & \equiv & dQ_{\vec{\alpha }}\left(\vec{A}\left({\vec{x}}_{\vec{\alpha }}\left(t\right),t\right)\cdot {\vec{v}}_{\vec{\alpha }}\left(t\right)-\phi \left({\vec{x}}_{\vec{\alpha }}\left(t\right),t\right)\right). \end{array}$$

The action and Lagrangian of the entire fluid are obtained by integrating over all possible values of $\vec{\alpha }$:

(3) $$\begin{array}{ccc} L & = & \int_{\vec{\alpha }}dL_{\vec{\alpha }}\\ A & = & \int_{\vec{\alpha }}dA_{\vec{\alpha }}=\int_{\vec{\alpha }}\int_{t1}^{t2}dL_{\vec{\alpha }}dt=\int_{t1}^{t2}\int_{\vec{\alpha }}dL_{\vec{\alpha }}dt=\int_{t1}^{t2}Ldt. \end{array}$$

A density is defined by dividing the quantity of a fluid element by its corresponding volume. This procedure is applied to the Lagrangian, mass, charge, and internal energy of each fluid element, using the following notation:

(4) $$L_{\vec{\alpha }}\equiv \frac{dL_{\vec{\alpha }}}{dV_{\vec{\alpha }}},\;\rho_{\vec{\alpha }}\equiv \frac{dM_{\vec{\alpha }}}{dV_{\vec{\alpha }}},\;\rho_{c\vec{\alpha }}\equiv \frac{dQ_{\vec{\alpha }}}{dV_{\vec{\alpha }}},\;e_{in\;\vec{\alpha }}\equiv \frac{dE_{in\;\vec{\alpha }}}{dV_{\vec{\alpha }}}$$

Each density-type quantity depends on the position $\vec{x}$ of the fluid element labeled $\vec{\alpha }$ at time *t*. For example,

(5) $$\rho \left(\vec{x},t\right)\equiv \rho \left({\vec{x}}_{\vec{\alpha }}\left(t\right),t\right)\equiv \rho_{\vec{\alpha }}\left(t\right).$$

We further define specific quantities by dividing a property of a fluid element by its mass. For instance, the specific internal energy $\varepsilon_{\vec{\alpha }}$ is given by

(6) $$\varepsilon_{\vec{\alpha }}\equiv \frac{dE_{in\;\vec{\alpha }}}{dM_{\vec{\alpha }}}\;\Rightarrow \;\rho_{\vec{\alpha }}\varepsilon_{\vec{\alpha }}=\frac{dM_{\vec{\alpha }}}{dV_{\vec{\alpha }}}\frac{dE_{in\;\vec{\alpha }}}{dM_{\vec{\alpha }}}=\frac{dE_{in\;\vec{\alpha }}}{dV_{\vec{\alpha }}}=e_{in\;\vec{\alpha }}$$

Therefore, the Lagrangian density can be decomposed as follows:

(7) $$\begin{array}{ccc} L_{\vec{\alpha }} & = & \frac{dL_{\vec{\alpha }}}{dV_{\vec{\alpha }}}=\frac{{dL}_{k\vec{\alpha }}}{dV_{\vec{\alpha }}}+\frac{{dL}_{i\vec{\alpha }}}{dV_{\vec{\alpha }}}-\frac{dE_{in\;\vec{\alpha }}}{dV_{\vec{\alpha }}}=L_{k\vec{\alpha }}+L_{i\vec{\alpha }}-e_{in\;\vec{\alpha }}\\ L_{k\vec{\alpha }} & \equiv & -\frac{\rho_{\vec{\alpha }}c^{2}}{\gamma_{\vec{\alpha }}}≃\frac{1}{2}\rho_{\vec{\alpha }}v_{\vec{\alpha }}{\left(t\right)}^{2}-\rho_{\vec{\alpha }}c^{2},\\ L_{i\vec{\alpha }} & \equiv & \rho_{c\vec{\alpha }}\left(\vec{A}\left({\vec{x}}_{\vec{\alpha }}\left(t\right),t\right)\cdot {\vec{v}}_{\vec{\alpha }}\left(t\right)-\varphi \left({\vec{x}}_{\vec{\alpha }}\left(t\right),t\right)\right). \end{array}$$

The fluid Lagrangian can therefore be expressed as a spatial integral:

(8) $$L=\int_{\vec{\alpha }}dL_{\vec{\alpha }}=\int_{\vec{\alpha }}L_{\vec{\alpha }}dV_{\vec{\alpha }}=\int L\left(\vec{x},t\right)d^{3}x.$$

This expression will be utilized in a later section dealing with the Eulerian representation of the fluid.

### 2.2. Variational Analysis

We now define the notation $\Delta \vec{x}\left(\vec{\alpha }\right)\equiv \vec{\xi }\left(\vec{\alpha }\right)$ to represent a variation in the trajectory ${\vec{x}}_{\vec{\alpha }}\left(t\right)$. Thus,

(9) $$\Delta {\vec{v}}_{\vec{\alpha }}\left(t\right)=\Delta \frac{d{\vec{x}}_{\vec{\alpha }}\left(t\right)}{dt}=\frac{d\Delta {\vec{x}}_{\vec{\alpha }}\left(t\right)}{dt}=\frac{d{\vec{\xi }}_{\vec{\alpha }}\left(t\right)}{dt}.$$

Moreover, according to Equation (9) of [25],

(10) $$\Delta \left(\frac{1}{\gamma_{\vec{\alpha }}}\right)=-\frac{\gamma_{\vec{\alpha }}{\vec{v}}_{\vec{\alpha }}\left(t\right)}{c^{2}}\frac{d{\vec{\xi }}_{\vec{\alpha }}\left(t\right)}{dt},\;\Delta \gamma_{\vec{\alpha }}=\frac{\gamma_{\vec{\alpha }}^{3}{\vec{v}}_{\vec{\alpha }}\left(t\right)}{c^{2}}\frac{d{\vec{\xi }}_{\vec{\alpha }}\left(t\right)}{dt}.$$

An ideal fluid is characterized by the property that each fluid element does not exchange mass, electric charge, or heat with neighboring elements. In variational form, this condition can be expressed as

(11) $$\Delta dM_{\vec{\alpha }}=\Delta dQ_{\vec{\alpha }}=\Delta dS_{\vec{\alpha }}=0.$$

From the perspective of thermodynamics, a change in the internal energy of a fluid element obeys the following relation in the rest frame of the element:

(12) $$\Delta dE_{in\;\vec{\alpha }0}=T_{\vec{\alpha }0}\Delta dS_{\vec{\alpha }0}-P_{\vec{\alpha }0}\Delta dV_{\vec{\alpha }0}.$$

In this expression, the first term represents the heating of the fluid element, while the second term corresponds to the work performed by the element on its neighbors. Here, $T_{\vec{\alpha }0}$ is the temperature of the fluid element in its rest frame, and $P_{\vec{\alpha }0}$ is its pressure. Since the rest mass of the fluid element remains constant and is independent of the reference frame, we can divide the equation by $dM_{\vec{\alpha }}$ to obtain the variation in the specific energy:

(13) $$\begin{array}{l} \Delta \varepsilon_{\vec{\alpha }0}\;=\;\Delta \frac{dE_{in\;\vec{\alpha }0}}{dM_{\vec{\alpha }}}=T_{\vec{\alpha }0}\Delta \frac{dS_{\vec{\alpha }0}}{dM_{\vec{\alpha }}}-P_{\vec{\alpha }0}\Delta \frac{dV_{\vec{\alpha }0}}{dM_{\vec{\alpha }}}\\ =\;T_{\vec{\alpha }0}\Delta s_{\vec{\alpha }0}-P_{\vec{\alpha }0}\Delta \frac{1}{\rho_{\vec{\alpha }0}}=T_{\vec{\alpha }0}\Delta s_{\vec{\alpha }0}+\frac{P_{\vec{\alpha }0}}{\rho_{\vec{\alpha }0}^{2}}\Delta \rho_{\vec{\alpha }0}.\;s_{\vec{\alpha }0}\equiv \frac{dS_{\vec{\alpha }0}}{dM_{\vec{\alpha }}} \end{array}$$

$s_{\vec{\alpha }0}$ is the specific entropy of the fluid element in its rest frame. It then follows that (we omit the index $\vec{\alpha }$ below)

(14) $$\frac{\partial \varepsilon_{0}}{\partial s_{0}}=T_{0},\;\frac{\partial \varepsilon_{0}}{\partial \rho_{0}}=\frac{P_{0}}{\rho_{0}^{2}}.$$

Another important thermodynamic quantity is the enthalpy, defined for a fluid element in its rest frame as

(15) $$dW_{\vec{\alpha }0}=dE_{in\;\vec{\alpha }0}+P_{\vec{\alpha }0}dV_{\vec{\alpha }0},$$

and the specific enthalpy

(16) $$w_{\vec{\alpha }0}=\frac{dW_{\vec{\alpha }0}}{dM_{\vec{\alpha }}}=\frac{dE_{in\;\vec{\alpha }0}}{dM_{\vec{\alpha }}}+P_{\vec{\alpha }0}\frac{dV_{\vec{\alpha }0}}{dM_{\vec{\alpha }}}=\varepsilon_{\vec{\alpha }0}+\frac{P_{\vec{\alpha }0}}{\rho_{\vec{\alpha }0}}.$$

By combining the above with Equation (14), we arrive at the following useful relations:

(17) $$w_{0}=\varepsilon_{0}+\frac{P_{0}}{\rho_{0}}=\varepsilon_{0}+\rho_{0}\frac{\partial \varepsilon_{0}}{\partial \rho_{0}}=\frac{\partial (\rho_{0}\varepsilon_{0})}{\partial \rho_{0}}.$$

(18) $$\frac{\partial w_{0}}{\partial \rho_{0}}=\frac{\partial (\varepsilon_{0}+\frac{P_{0}}{\rho_{0}})}{\partial \rho_{0}}=-\frac{P_{0}}{\rho_{0}^{2}}+\frac{1}{\rho_{0}}\frac{\partial P_{0}}{\partial \rho_{0}}+\frac{\partial \varepsilon_{0}}{\partial \rho_{0}}=-\frac{P_{0}}{\rho_{0}^{2}}+\frac{1}{\rho_{0}}\frac{\partial P_{0}}{\partial \rho_{0}}+\frac{P_{0}}{\rho_{0}^{2}}=\frac{1}{\rho_{0}}\frac{\partial P_{0}}{\partial \rho_{0}}.$$

(19) $$\vec{\nabla }w_{0}=T_{0}\vec{\nabla }s_{0}+\frac{1}{\rho_{0}}\vec{\nabla }P_{0}.$$

For an ideal fluid, heat conduction and radiation are neglected, so only convection is taken into account. Therefore, $\Delta dS_{\vec{\alpha }0}=0$ and it follows that:

(20) $$\Delta dE_{in\;\vec{\alpha }0}=-P_{0}\Delta dV_{\vec{\alpha }0}.$$

We now establish some relationships between the rest frame of a fluid element and any other frame in which the element is moving (often referred to as the “laboratory” frame). First, we note that, in the rest frame, the velocity is zero (by definition), so, according to Equation (9) of [25],

(21) $$d\tau =cdt_{0}=cdt\sqrt{1-\frac{v^{2}}{c^{2}}}=\frac{cdt}{\gamma }\;\Rightarrow \;dt_{0}=\frac{dt}{\gamma }.$$

It is a well-known fact that the four-volume is Lorentz-invariant, so that

(22) $$dV_{0}dt_{0}=dVdt=dVdt_{0}\gamma ,\;\Rightarrow dV_{0}=\gamma dV.$$

Thus,

(23) $$\rho_{0}=\frac{dM}{dV_{0}}=\frac{1}{\gamma }\frac{dM}{dV}=\frac{\rho }{\gamma },\;\Rightarrow \;\rho =\gamma \rho_{0}.$$

Furthermore, the action given in Equation (1) is Lorentz-invariant, which implies

(24) $$\begin{array}{cc} & dE_{in\;\vec{\alpha }0}dt_{0}=dE_{in\;\vec{\alpha }}dt=dE_{in\;\vec{\alpha }}dt_{0}\gamma \\ \Rightarrow & dE_{in\;\vec{\alpha }0}=\gamma dE_{in\;\vec{\alpha }},\;dE_{in\;\vec{\alpha }}=\frac{dE_{in\;\vec{\alpha }0}}{\gamma } \end{array}$$

We can now consider a variation of the internal energy of a fluid element:

(25) $$\Delta dE_{in\;\vec{\alpha }}=\Delta \left(\frac{1}{\gamma }\right)dE_{in\;\vec{\alpha }0}+\frac{1}{\gamma }\Delta dE_{in\;\vec{\alpha }0}.$$

Thus, according to Equations (20) and (22), it follows that

(26) $$\Delta dE_{in\;\vec{\alpha }}=\Delta \left(\frac{1}{\gamma }\right)dE_{in\;\vec{\alpha }0}-\frac{1}{\gamma }P_{0}\Delta dV_{\vec{\alpha }0}=\Delta \left(\frac{1}{\gamma }\right)dE_{in\;\vec{\alpha }0}-\frac{1}{\gamma }P_{0}\Delta \left(\gamma dV_{\vec{\alpha }}\right).$$

Taking into account the enthalpy quantity defined in Equation (15), it follows that

(27) $$\Delta dE_{in\;\vec{\alpha }}=\Delta \left(\frac{1}{\gamma }\right)\left(dE_{in\;\vec{\alpha }0}+P_{0}dV_{\vec{\alpha }0}\right)-\;P_{0}\Delta dV_{\vec{\alpha }}=\Delta \left(\frac{1}{\gamma }\right)dW_{\vec{\alpha }0}-\;P_{0}\Delta dV_{\vec{\alpha }}.$$

We shall now vary the fluid element’s volume. At some specific time *t*, the fluid element’s volume is

(28) $$dV_{\vec{\alpha },t}=d^{3}x\left(\vec{\alpha },t\right)$$

The Jacobian relates the fluid element’s current volume to its volume at t=0:

(29) $$d^{3}x\left(\vec{\alpha },t\right)=Jd^{3}x\left(\vec{\alpha },0\right),\;J\equiv {\vec{\nabla }}_{0}x_{1}\cdot \left({\vec{\nabla }}_{0}x_{2}\times {\vec{\nabla }}_{0}x_{3}\right)$$

${\vec{\nabla }}_{0}$ is calculated with respect to the fluid element’s coordinates at t=0:

(30) $${\vec{\nabla }}_{0}\equiv \left(\frac{\partial }{\partial x{\left(\vec{\alpha },0\right)}_{1}},\frac{\partial }{\partial x{\left(\vec{\alpha },0\right)}_{2}},\frac{\partial }{\partial x{\left(\vec{\alpha },0\right)}_{3}}\right).$$

An interesting observation is that building a spatial volume element from three vectors, using a Jacobian as in Equation (29), closely resembles the construction of a four-dimensional volume element from four scalars, where one also considers the Jacobian of the transformation from spacetime coordinates to scalar field space, as was described long ago in [27,28,29,30]. Thus,

(31) $$\begin{array}{ccc} \Delta dV_{\vec{\alpha },t} & = & \Delta d^{3}x\left(\vec{\alpha },t\right)=\Delta J\;d^{3}x\left(\vec{\alpha },0\right)=\frac{\Delta J}{J}d^{3}x\left(\vec{\alpha },t\right)=\frac{\Delta J}{J}dV_{\vec{\alpha },t},\\ (\Delta d^{3}x\left(\vec{\alpha },0\right) & = & 0). \end{array}$$

We calculate the variation in *J* as follows:

(32) $$\begin{array}{ccc} \Delta J & = & {\vec{\nabla }}_{0}\Delta x_{1}\cdot \left({\vec{\nabla }}_{0}x_{2}\times {\vec{\nabla }}_{0}x_{3}\right)+{\vec{\nabla }}_{0}x_{1}\cdot \left({\vec{\nabla }}_{0}\Delta x_{2}\times {\vec{\nabla }}_{0}x_{3}\right)\\ & + & {\vec{\nabla }}_{0}x_{1}\cdot \left({\vec{\nabla }}_{0}x_{2}\times {\vec{\nabla }}_{0}\Delta x_{3}\right), \end{array}$$

Thus,

(33) $$\begin{array}{c} {\vec{\nabla }}_{0}\Delta x_{1}\cdot \left({\vec{\nabla }}_{0}x_{2}\times {\vec{\nabla }}_{0}x_{3}\right)={\vec{\nabla }}_{0}\xi_{1}\cdot \left({\vec{\nabla }}_{0}x_{2}\times {\vec{\nabla }}_{0}x_{3}\right)\\ \;=\;\partial_{k}\xi_{1}{\vec{\nabla }}_{0}x_{k}\cdot \left({\vec{\nabla }}_{0}x_{2}\times {\vec{\nabla }}_{0}x_{3}\right)=\partial_{1}\xi_{1}{\vec{\nabla }}_{0}x_{1}\cdot \left({\vec{\nabla }}_{0}x_{2}\times {\vec{\nabla }}_{0}x_{3}\right)=\partial_{1}\xi_{1}J.\\ {\vec{\nabla }}_{0}x_{1}\cdot \left({\vec{\nabla }}_{0}\Delta x_{2}\times {\vec{\nabla }}_{0}x_{3}\right)={\vec{\nabla }}_{0}x_{1}\cdot \left({\vec{\nabla }}_{0}\xi_{2}\times {\vec{\nabla }}_{0}x_{3}\right)\\ \;=\;\partial_{k}\xi_{2}{\vec{\nabla }}_{0}x_{1}\cdot \left({\vec{\nabla }}_{0}x_{k}\times {\vec{\nabla }}_{0}x_{3}\right)=\partial_{2}\xi_{2}{\vec{\nabla }}_{0}x_{1}\cdot \left({\vec{\nabla }}_{0}x_{2}\times {\vec{\nabla }}_{0}x_{3}\right)=\partial_{2}\xi_{2}J.\\ {\vec{\nabla }}_{0}x_{1}\cdot \left({\vec{\nabla }}_{0}x_{2}\times {\vec{\nabla }}_{0}\Delta x_{3}\right)={\vec{\nabla }}_{0}x_{1}\cdot \left({\vec{\nabla }}_{0}x_{2}\times {\vec{\nabla }}_{0}\xi_{3}\right)\\ \;=\;\partial_{k}\xi_{3}{\vec{\nabla }}_{0}x_{1}\cdot \left({\vec{\nabla }}_{0}x_{2}\times {\vec{\nabla }}_{0}x_{k}\right)=\partial_{3}\xi_{3}{\vec{\nabla }}_{0}x_{1}\cdot \left({\vec{\nabla }}_{0}x_{2}\times {\vec{\nabla }}_{0}x_{3}\right)=\partial_{3}\xi_{3}J. \end{array}$$

This leads to

(34) $$\Delta J=\partial_{1}\xi_{1}J+\partial_{2}\xi_{2}J+\partial_{3}\xi_{3}J=\vec{\nabla }\cdot \vec{\xi }\;J,\;\Delta dV_{\vec{\alpha },t}=\vec{\nabla }\cdot \vec{\xi }\;dV_{\vec{\alpha },t}.$$

Thus, we derive the complete variation in the internal energy given in Equation (27) in the form

(35) $$\Delta dE_{in\;\vec{\alpha }}=\Delta \left(\frac{1}{\gamma }\right)dW_{\vec{\alpha }0}-P_{0}\vec{\nabla }\cdot \vec{\xi }\;dV_{\vec{\alpha },t}.$$

If we consider also Equation (10), the following result is obtained:

(36) $$\Delta dE_{in\;\vec{\alpha }}=-P_{\vec{\alpha }0}\vec{\nabla }\cdot {\vec{\xi }}_{\vec{\alpha }}\;dV_{\vec{\alpha },t}-\frac{\gamma_{\vec{\alpha }}{\vec{v}}_{\vec{\alpha }}\left(t\right)}{c^{2}}dW_{\vec{\alpha }0}\cdot \frac{d{\vec{\xi }}_{\vec{\alpha }}\left(t\right)}{dt}.$$

The variation in internal energy is the only new aspect compared to the calculation for a previously described system of particles; the remainder of the analysis is straightforward. By varying Equation (1), we therefore obtain

(37) $$\begin{array}{ccc} \Delta dA_{\vec{\alpha }} & = & \int_{t1}^{t2}\Delta dL_{\vec{\alpha }}dt,\;\Delta dL_{\vec{\alpha }}=\Delta dL_{k\vec{\alpha }}+\Delta dL_{i\vec{\alpha }}-\Delta dE_{in\;\vec{\alpha }}\\ \Delta dL_{k\vec{\alpha }} & = & -dM_{\vec{\alpha }}c^{2}\Delta \left(\frac{1}{\gamma_{\vec{\alpha }}}\right)=dM_{\vec{\alpha }}\gamma_{\vec{\alpha }}{\vec{v}}_{\vec{\alpha }}\left(t\right)\cdot \frac{d{\vec{\xi }}_{\vec{\alpha }}\left(t\right)}{dt},\\ \Delta dL_{i\vec{\alpha }} & = & dQ_{\vec{\alpha }}\left(\Delta \vec{A}\left({\vec{x}}_{\vec{\alpha }}\left(t\right),t\right)\cdot {\vec{v}}_{\vec{\alpha }}\left(t\right)+\vec{A}\left({\vec{x}}_{\vec{\alpha }}\left(t\right),t\right)\cdot \Delta {\vec{v}}_{\vec{\alpha }}\left(t\right)\right.\\ & - & \left.\Delta \phi ({\vec{x}}_{\vec{\alpha }}\left(t\right),t)\right). \end{array}$$

Subtracting the internal and kinetic parts of the varied Lagrangian while taking into account the specific enthalpy defined in Equation (16), the following result is derived:

(38) $$\Delta dL_{k\vec{\alpha }}-\Delta dE_{in\;\vec{\alpha }}=dM_{\vec{\alpha }}\gamma_{\vec{\alpha }}\left(1+\frac{w_{0}}{c^{2}}\right){\vec{v}}_{\vec{\alpha }}\left(t\right)\cdot \frac{d{\vec{\xi }}_{\vec{\alpha }}\left(t\right)}{dt}+P_{\vec{\alpha }0}\vec{\nabla }\cdot {\vec{\xi }}_{\vec{\alpha }}\;dV_{\vec{\alpha },t}.$$

The electromagnetic-related variation terms are not different from Equations (A47) and (A48) of [1], and their derivation will not be repeated here. Denoting the Lorentz force that is suffered by particle $\vec{\alpha }$ as

(39) $$d{\vec{F}}_{L\vec{\alpha }}\equiv dQ_{\vec{\alpha }}\left[{\vec{v}}_{\vec{\alpha }}\times \vec{B}\left({\vec{x}}_{\vec{\alpha }}\left(t\right),t\right)+\vec{E}\left({\vec{x}}_{\vec{\alpha }}\left(t\right),t\right)\right],$$

we obtain

(40) $$\Delta dL_{i\vec{\alpha }}=\frac{d(dQ_{\vec{\alpha }}\vec{A}\left({\vec{x}}_{\vec{\alpha }}\left(t\right),t\right)\cdot {\vec{\xi }}_{\vec{\alpha }})}{dt}+d{\vec{F}}_{L\vec{\alpha }}\cdot {\vec{\xi }}_{\vec{\alpha }}.$$

Let us introduce the shorthand notation

(41) $$\bar{\lambda }\equiv 1+\frac{w_{0}}{c^{2}},\;\lambda \equiv \gamma \bar{\lambda }=\gamma \left(1+\frac{w_{0}}{c^{2}}\right).$$

Provided that the enthalpy of a fluid element in its frame of rest is minute with respect to its rest energy, i.e.,

(42) $$dW_{\vec{\alpha }0}\ll dM_{\vec{\alpha }}c^{2}\;\Rightarrow \;1\gg \frac{dW_{\vec{\alpha }0}}{dM_{\vec{\alpha }}c^{2}}=\frac{w_{\vec{\alpha }0}}{c^{2}},$$

it follows that, in the classical limit (with limitations on both the enthalpy of the fluid element and its speed), we will have

(43) $$\bar{\lambda }≃1,\;\lambda ≃1.$$

The varied action of a single relativistic fluid element is thus

(44) $$\begin{array}{c} \Delta dA_{\vec{\alpha }}=\int_{t1}^{t2}\Delta dL_{\vec{\alpha }}dt={\left.\left(dM_{\vec{\alpha }}\lambda_{\vec{\alpha }}{\vec{v}}_{\vec{\alpha }}\left(t\right)+dQ_{\vec{\alpha }}\vec{A}\left({\vec{x}}_{\vec{\alpha }}\left(t\right),t\right)\right)\cdot {\vec{\xi }}_{\vec{\alpha }}\right|}_{t1}^{t2}\\ -\int_{t1}^{t2}\left(dM_{\vec{\alpha }}\frac{d(\lambda_{\vec{\alpha }}{\vec{v}}_{\vec{\alpha }}\left(t\right))}{dt}\cdot {\vec{\xi }}_{\vec{\alpha }}-d{\vec{F}}_{L\vec{\alpha }}\cdot {\vec{\xi }}_{\vec{\alpha }}-P_{\vec{\alpha }0}\vec{\nabla }\cdot {\vec{\xi }}_{\vec{\alpha }}\;dV_{\vec{\alpha },t}\right)dt. \end{array}$$

It follows that the variation in the entire relativistic fluid action can be integrated as follows:

(45) $$\begin{array}{c} \Delta A=\Delta \int_{\vec{\alpha }}dA_{\vec{\alpha }}={\left.\int_{\vec{\alpha }}\left(dM_{\vec{\alpha }}\lambda_{\vec{\alpha }}{\vec{v}}_{\vec{\alpha }}\left(t\right)+dQ_{\vec{\alpha }}\vec{A}\left(\vec{x}\left(\vec{\alpha },t\right),t\right)\right)\cdot {\vec{\xi }}_{\vec{\alpha }}\right|}_{t1}^{t2}\\ -\int_{t1}^{t2}\int_{\vec{\alpha }}\left(dM_{\vec{\alpha }}\frac{d(\lambda_{\vec{\alpha }}{\vec{v}}_{\vec{\alpha }}\left(t\right))}{dt}\cdot {\vec{\xi }}_{\vec{\alpha }}-d{\vec{F}}_{L\vec{\alpha }}\cdot {\vec{\xi }}_{\vec{\alpha }}-P_{\vec{\alpha }0}\vec{\nabla }\cdot {\vec{\xi }}_{\vec{\alpha }}\;dV_{\vec{\alpha }}\right)dt. \end{array}$$

From Equation (4), we obtain

(46) $$dM_{\vec{\alpha }}=\rho_{\vec{\alpha }}\;dV_{\vec{\alpha }},\;dQ_{\vec{\alpha }}=\rho_{c\vec{\alpha }}\;dV_{\vec{\alpha }}$$

Using the above equality relations, the $\vec{\alpha }$ integral becomes a volume integral, and we are able to write the variation in the fluid action in the form (we omit $\vec{\alpha }$)

(47) $$\Delta A={\left.\int \left(\rho \lambda \vec{v}+\rho_{c}\vec{A}\right)\cdot \vec{\xi }dV\right|}_{t1}^{t2}-\int_{t1}^{t2}\int \left(\rho \frac{d(\lambda \vec{v})}{dt}\cdot \vec{\xi }-{\vec{f}}_{L}\cdot \vec{\xi }-P_{0}\vec{\nabla }\cdot \vec{\xi }\right)dVdt,$$

in which we use the following Lorentz force density definition:

(48) $${\vec{f}}_{L\vec{\alpha }}\equiv \frac{d{\vec{F}}_{L\vec{\alpha }}}{dV_{\vec{\alpha }}}=\rho_{c\vec{\alpha }}\left[{\vec{v}}_{\vec{\alpha }}\times \vec{B}\left({\vec{x}}_{\vec{\alpha }}\left(t\right),t\right)+\vec{E}\left({\vec{x}}_{\vec{\alpha }}\left(t\right),t\right)\right].$$

Taking into account that

(49) $$P_{0}\vec{\nabla }\cdot \vec{\xi }=\vec{\nabla }\cdot \left(P_{0}\vec{\xi }\right)-\vec{\xi }\cdot \vec{\nabla }P_{0},$$

and taking advantage of the theorem of Gauss, the action’s variation takes the form

(50) $$\begin{array}{ccc} \Delta A & = & {\left.\int \left(\rho \lambda \vec{v}+\rho_{c}\vec{A}\right)\cdot \vec{\xi }dV\right|}_{t1}^{t2}\\ & - & \int_{t1}^{t2}\left[\int \left(\rho \frac{d(\lambda \vec{v})}{dt}-{\vec{f}}_{L}+\vec{\nabla }P_{0}\right)\cdot \vec{\xi }dV-\oint P_{0}\vec{\xi }\cdot d\vec{\Sigma }\right]dt. \end{array}$$

It follows that, if the variation in the action is zero for a displacement field $\vec{\xi }$ satisfying $\vec{\xi }\left(t_{1}\right)=\vec{\xi }\left(t_{2}\right)=0$ and vanishing on the boundary surface enclosing the fluid, the Euler equation for a relativistic charged fluid holds, namely

(51) $$\frac{d(\lambda \vec{v})}{dt}=-\frac{\vec{\nabla }P_{0}}{\rho }+\frac{{\vec{f}}_{L}}{\rho },\;\frac{d\vec{v}}{dt}≃-\frac{\vec{\nabla }P_{0}}{\rho }+\frac{{\vec{f}}_{L}}{\rho },$$

In the specific case wherein each fluid element consists of identical microscopic particles with mass *m* and charge *e*, the mass and charge densities are directly proportional to the particle number density *n*:

(52) $$\rho =m\;n,\;\rho_{c}=e\;n\Rightarrow \frac{{\vec{f}}_{L}}{\rho }=k\left[\vec{v}\times \vec{B}+\vec{E}\right],k\equiv \frac{e}{m}$$

Therefore, aside from the contributions associated with the internal energy, the equation has the same structure as that of a point particle. In the case of a neutral fluid, it takes the form

(53) $$\frac{d(\lambda \vec{v})}{dt}=-\frac{\vec{\nabla }P_{0}}{\rho },\;\frac{d\vec{v}}{dt}≃-\frac{\vec{\nabla }P_{0}}{\rho }.$$

Certain authors choose to express the preceding equation using the energy of each fluid element per unit volume in the rest frame, which combines the internal energy and the rest mass contributions:

(54) $$e_{0}\equiv \rho_{0}c^{2}+\rho_{0}\varepsilon_{0}.$$

It is easy to show that

(55) $$\bar{\lambda }=1+\frac{w_{0}}{c^{2}}=\frac{e_{0}+P_{0}}{\rho_{0}c^{2}}.$$

By applying the above relation and performing a few algebraic steps, we can express Equation (53) in a form that some authors may find more convenient:

(56) $$\left(e_{0}+P_{0}\right)\frac{\gamma }{c^{2}}\frac{d(\gamma \vec{v})}{dt}=-\vec{\nabla }P_{0}-\frac{\gamma^{2}}{c^{2}}\frac{dP_{0}}{dt}\vec{v}.$$

In practical fluid dynamics, the fluid is characterized using local, measurable quantities rather than those associated with invisible infinitesimal “fluid elements”. This approach is known as the Eulerian description, where the formulation is based on flow fields instead of following individual fluid elements, as will be explained later. Before introducing the Eulerian framework, it is useful to briefly discuss the canonical energy–momentum tensor in general and its specific form within the relativistic Lagrangian description of fluid flows.

## 3. Canonical Energy–Momentum Tensor

Let us consider a general field of spacetime of *K* components $\eta_{a}\left(x^{\mu }\right)$ and let us consider a Lagrangian density of the form

(57) $$L=L\left(\eta_{a},\partial_{\mu }\eta_{a}\right),\;\partial_{\mu }\equiv \frac{\partial }{\partial x^{\mu }}.$$

The raising and lowering of indices will be performed as usual with the Lorentz–Minkowski metric $\eta_{\mu \nu }\equiv \mathrm{diag}\left(+1,-1,-1,-1\right)$. Provided that we are given the action

(58) $$A\equiv \int d^{4}xL$$

it will take an extremum, δA=0, if $\eta_{a}\left(x^{\mu }\right)$ satisfies the Euler–Lagrange equations:

(59) $$\frac{d}{dx_{\nu }}\left(\frac{\partial L}{\partial (\partial_{\nu }\eta_{a})}\right)=\frac{\partial L}{\partial \eta_{a}}.$$

In the above, $\frac{d}{dx_{\nu }}$ is the total derivative with respect to the coordinate $x_{\nu }$, i.e., one takes into account the dependence of $\eta_{a}$ on the coordinates. Moreover, Einstein summation convention is assumed. The above is correct provided that the appropriate initial and final time conditions, as well as boundary conditions, are satisfied.

Then, one can define an energy–momentum tensor of the form [20]

(60) $$T_{\mu \nu }\equiv \partial_{\mu }\eta_{a}\frac{\partial L}{\partial (\partial^{\nu }\eta_{a})}-\eta_{\mu \nu }L.$$

In the above, we assume Einstein summation convention with respect to the scalar field index *a*. This quantity will satisfy, due to Euler–Lagrange Equation (59), the following conservation laws:

(61) $$\frac{d}{dx_{\nu }}T_{\mu \nu }=0$$

The quantities

(62) $${\bar{P}}_{\mu }=\int d^{3}xT_{\mu 0}$$

are conserved in time, i.e.,

(63) $$\frac{d{\bar{P}}_{\mu }}{dt}=0.$$

${\bar{P}}_{0}$ denotes the system’s total energy, and ${\bar{P}}_{i}$ denotes the components of the total linear momentum of the system i∈[1,2,3].

For a Lagrangian fluid, there is a dependence of one coordinate *t* ($x^{0}=ct$); however, the field indices are continuous (through the $\vec{\alpha }$ label) and therefore infinite. It follows that

(64) $$T_{L00}=\int_{\vec{\alpha }}\left[\frac{d{\vec{x}}_{\vec{\alpha }}\left(t\right)}{dt}\cdot \frac{\partial L}{\partial (\frac{d{\vec{x}}_{\vec{\alpha }}\left(t\right)}{dt})}\right]-L=\int_{\vec{\alpha }}\left[{\vec{v}}_{\vec{\alpha }}\cdot \frac{\partial L}{\partial {\vec{v}}_{\vec{\alpha }}}\right]-L.$$

Now, according to Equation (3),

(65) $$\frac{\partial L}{\partial {\vec{v}}_{\vec{\alpha }}}=\frac{\partial \int_{{\vec{\alpha }}^{\prime }}dL_{{\vec{\alpha }}^{\prime }}}{\partial {\vec{v}}_{\vec{\alpha }}}=\frac{\partial dL_{\vec{\alpha }}}{\partial {\vec{v}}_{\vec{\alpha }}}.$$

Thus, it follows from Equation (2) that

(66) $$\frac{\partial L}{\partial {\vec{v}}_{\vec{\alpha }}}=\frac{\partial dL_{k\vec{\alpha }}}{\partial {\vec{v}}_{\vec{\alpha }}}+\frac{\partial dL_{i\vec{\alpha }}}{\partial {\vec{v}}_{\vec{\alpha }}}-\frac{\partial dE_{in\;\vec{\alpha }}}{\partial {\vec{v}}_{\vec{\alpha }}}.$$

Using the definitions of $dL_{k\vec{\alpha }},dL_{i\vec{\alpha }},dE_{in\;\vec{\alpha }}$ given in Equation (2), and the fact that $dE_{in\;\vec{\alpha }}$ is velocity-dependent according to Equation (24), it follows that

(67) $$\frac{\partial L}{\partial {\vec{v}}_{\vec{\alpha }}}=dM_{\vec{\alpha }}\gamma_{\vec{\alpha }}{\vec{v}}_{\vec{\alpha }}+dQ_{\vec{\alpha }}{\vec{A}}_{\vec{\alpha }}+dE_{in\;\vec{\alpha }0}\frac{\gamma_{\vec{\alpha }}}{c^{2}}{\vec{v}}_{\vec{\alpha }},$$

in which we have used the identity

(68) $$\frac{\partial \gamma_{\vec{\alpha }}^{-1}}{\partial {\vec{v}}_{\vec{\alpha }}}=-\frac{\gamma_{\vec{\alpha }}}{c^{2}}{\vec{v}}_{\vec{\alpha }}.$$

Plugging Equation (68) into Equation (64) will lead, after some simplification, to the form

(69) $$T_{L00}=\int_{\vec{\alpha }}\left[dM_{\vec{\alpha }}c^{2}\gamma_{\vec{\alpha }}+dQ_{\vec{\alpha }}\phi \left({\vec{x}}_{\vec{\alpha }}\right)+dE_{in\;\vec{\alpha }0}\gamma_{\vec{\alpha }}\right].$$

This can be written as a volume integral:

(70) $$T_{L00}=\int d^{3}x\left[\rho \left(c^{2}+\varepsilon_{0}\right)\gamma +\rho_{c}\phi \right].$$

The above expression denotes the fluid’s total energy, which is constant in time.

## 4. Action Principle of Eulerian Relativistic Charged Fluids

In what follows, we adopt the derivation presented in [1,2,31], but now we include the relativistic character of the flow. This requires constructing an action that remains invariant under Lorentz transformations. Let us therefore consider the action

(71) $$\begin{array}{ccc} A & \equiv & \int Ld^{3}xdt,\;L\equiv L_{0}+L_{2}+L_{i}\\ L_{0} & \equiv & -\rho \left(\frac{c^{2}}{\gamma }+\varepsilon \right)=-\rho_{0}\left(c^{2}+\varepsilon_{0}\right)=-e_{0},\\ L_{2} & \equiv & \nu \partial^{\nu }\left(\rho_{0}u_{\nu }\right)-\rho_{0}\alpha u_{\nu }\partial^{\nu }\beta -\rho_{0}\sigma u_{\nu }\partial^{\nu }s,\\ L_{i} & \equiv & -\rho_{c}A^{\nu }v_{\nu },\;v_{\nu }\equiv \frac{dx_{\nu }}{dt}. \end{array}$$

We thus obtain the limit of small velocities as follows:

(72) $$L_{0}≃\rho \left(\frac{1}{2}v^{2}-\varepsilon -c^{2}\right)$$

It is easy to show that

(73) $$u_{\mu }=\gamma \left(c,\vec{v}\right).$$

Taking into account Equation (23), we write the relativistic Lagrangian densities in a spacetime formalism:

(74) $$L_{2}=\nu \left[\frac{\partial \rho }{\partial t}+\vec{\nabla }\cdot \left(\rho \vec{v}\right)\right]-\rho \alpha \frac{d\beta }{dt}-\rho \sigma \frac{ds}{dt}\;L_{i}=\rho_{c}\left(\vec{A}\cdot \vec{v}-\phi \right).$$

In this formulation, the variational quantities are treated as space- and time-dependent fields. These consist of the velocity vector field $\vec{v}\left(\vec{x},t\right)$ and the scalar density field $\rho (\vec{x},t)$. In this context, we employ the material derivative, which can be expressed as

(75) $$\frac{dg(\vec{\alpha },t)}{dt}=\frac{dg(\vec{x}\left(\vec{\alpha },t\right),t)}{dt}=\frac{\partial g}{\partial t}+\frac{d\vec{x}}{dt}\cdot \vec{\nabla }g=\frac{\partial g}{\partial t}+\vec{v}\cdot \vec{\nabla }g$$

Once *g* is treated as a function of $\vec{x}$ and *t* instead of a function of $\vec{\alpha }$ and *t*, quantities that are straightforwardly conserved in the Lagrangian approach—such as labels, mass, charge, and entropy—can be incorporated into this formalism by introducing Lagrange multipliers ν, α, and σ to enforce the corresponding constraints:

(76) $$\begin{array}{c} \frac{\partial \rho }{\partial t}+\vec{\nabla }\cdot \left(\rho \vec{v}\right)=0\\ \frac{d\beta }{dt}=0\\ \frac{ds}{dt}=0. \end{array}$$

Assuming ρ≠0, these conditions include the continuity equation, which guarantees mass conservation; the requirement that β acts as a label (i.e., a comoving quantity); and the conservation of the specific entropy *s*. Varying the action with respect to β and using the continuity equation (lagmulb) leads to an equation analogous to the derivation of Equations (67)–(69) in [2]:

(77) $$\frac{d\alpha }{dt}=0$$

Thus, for ρ≠0, both α and β serve as labels. The specific internal energy $\varepsilon_{0}$, defined in Equation (6), depends on the fluid’s thermodynamic properties and is specified through an equation of state as a function of the density and specific entropy. We assume that the entropy of a fluid element is Lorentz-invariant, meaning that $dS_{\vec{\alpha }0}=dS_{\vec{\alpha }}$, and therefore $s_{\vec{\alpha }0}=s_{\vec{\alpha }}$. Varying the action with respect to the specific entropy *s* then gives

(78) $$\frac{d\sigma }{dt}=\frac{T_{0}}{\gamma },$$

assuming that the relevant boundary conditions are fulfilled. The electromagnetic potentials $\vec{A}$ and ϕ are treated as fixed functions of the coordinates and are not varied. We also assume that each fluid element consists of microscopic particles with mass *m* and charge *e*, so it follows from Equation (52) that

(79) $$\rho_{c}=k\rho .$$

Taking a variational derivative with respect to ρ, we derive

(80) $$\begin{array}{ccc} \delta_{\rho }A & = & \int d^{3}xdt\delta \rho \left[-\frac{c^{2}}{\gamma }-w_{0}\frac{\delta \rho_{0}}{\delta \rho }-\frac{\partial \nu }{\partial t}-\vec{v}\cdot \vec{\nabla }\nu +k\left(\vec{A}\cdot \vec{v}-\phi \right)\right]\\ & + & \oint d\vec{S}\cdot \vec{v}\delta \rho \nu +\int d\vec{\Sigma }\cdot \vec{v}\delta \rho \left[\nu \right]+\int d^{3}{x\nu \delta \rho |}_{t_{0}}^{t_{1}} \end{array}$$

or

(81) $$\begin{array}{ccc} \delta_{\rho }A & = & \int d^{3}xdt\delta \rho \left[-\frac{c^{2}+w_{0}}{\gamma }-\frac{\partial \nu }{\partial t}-\vec{v}\cdot \vec{\nabla }\nu +k\left(\vec{A}\cdot \vec{v}-\phi \right)\right]\\ & + & \oint d\vec{S}\cdot \vec{v}\delta \rho \nu +\int d\vec{\Sigma }\cdot \vec{v}\delta \rho \left[\nu \right]+\int d^{3}{x\nu \delta \rho |}_{t_{0}}^{t_{1}} \end{array}$$

Provided that δρ disappears on the spatial and temporal boundaries and the cut, we derive

(82) $$\frac{d\nu }{dt}=\frac{\partial \nu }{\partial t}+\vec{v}\cdot \vec{\nabla }\nu =-\frac{c^{2}+w_{0}}{\gamma }+k\left(\vec{A}\cdot \vec{v}-\phi \right)\Rightarrow \frac{d\nu }{dt}≃\frac{1}{2}v^{2}-w_{0}-c^{2}+k\left(\vec{A}\cdot \vec{v}-\phi \right).$$

Equation (82) can be written compactly as

(83) $$\frac{d\nu }{dt}=-c^{2}\frac{\bar{\lambda }}{\gamma }+k\left(\vec{A}\cdot \vec{v}-\phi \right)=-c^{2}\frac{\lambda }{\gamma^{2}}+k\left(\vec{A}\cdot \vec{v}-\phi \right),$$

and $\bar{\lambda }$ is introduced in Equation (41). As a final step, the action is varied with respect to the velocity $\vec{v}$, taking into account the identity

(84) $$\delta_{\vec{v}}\frac{1}{\gamma }=-\gamma \frac{\vec{v}\cdot \delta \vec{v}}{c^{2}}$$

We obtain the following result:

(85) $$\begin{array}{ccc} \delta_{\vec{v}}A & = & \int d^{3}xdt\rho \delta \vec{v}\cdot \left[\gamma \vec{v}-\frac{w_{0}}{\rho }\frac{\delta \rho_{0}}{\delta \vec{v}}-\vec{\nabla }\nu -\alpha \vec{\nabla }\beta -\sigma \vec{\nabla }s+k\vec{A}\right]\\ & + & \oint d\vec{S}\cdot \delta \vec{v}\rho \nu +\int d\vec{\Sigma }\cdot \delta \vec{v}\rho \left[\nu \right]. \end{array}$$

However,

(86) $$\frac{\delta \rho_{0}}{\delta \vec{v}}=\rho \frac{\delta \frac{1}{\gamma }}{\delta \vec{v}}=-\rho \gamma \frac{\vec{v}}{c^{2}}$$

Using λ defined in Equation (41), we may write

(87) $$\begin{array}{ccc} \delta_{\vec{v}}A & = & \int d^{3}xdt\rho \delta \vec{v}\cdot \left[\lambda \vec{v}-\vec{\nabla }\nu -\alpha \vec{\nabla }\beta -\sigma \vec{\nabla }s+k\vec{A}\right]\\ & + & \oint d\vec{S}\cdot \delta \vec{v}\rho \nu +\int d\vec{\Sigma }\cdot \delta \vec{v}\rho \left[\nu \right]. \end{array}$$

The boundary terms above include an integral over the external boundary, $\oint d\vec{S}$, and an integral over a cut, $\int d\vec{\Sigma }$, which is needed if ν is not single-valued. The external boundary term vanishes in situations such as astrophysical flows where ρ=0 at the free-flow boundary or when the fluid is contained in a vessel imposing a no-flux condition, $\delta \vec{v}\cdot \hat{n}=0$, with $\hat{n}$ the unit normal to the boundary. The cut term vanishes if the velocity variations are parallel to the cut, satisfying a Kutta-type condition. When the boundary terms vanish, the velocity field $\vec{v}$ must take the following form:

(88) $$\lambda \vec{v}=\alpha \vec{\nabla }\beta +\sigma \vec{\nabla }s+\vec{\nabla }\nu -k\vec{A},\Rightarrow \vec{v}≃\alpha \vec{\nabla }\beta +\sigma \vec{\nabla }s+\vec{\nabla }\nu -k\vec{A}.$$

This represents a generalization of the Clebsch representation for a non-barotropic flow field (see, e.g., [6] and (page 248, [32])) extended to a relativistic charged flow. We note that the function ν contributes to the velocity field only through its gradient, which implies that adding an arbitrary function of time to ν does not change the physical velocity field. By redefining

(89) $$\bar{\nu }\equiv \nu +c^{2}t,$$

we derive the more classical form of Equations (82) and (88):

(90) $$\frac{d\bar{\nu }}{dt}≃\frac{1}{2}v^{2}-w_{0}+k\left(\vec{A}\cdot \vec{v}-\phi \right),\;\vec{v}≃\alpha \vec{\nabla }\beta +\sigma \vec{\nabla }s+\vec{\nabla }\bar{\nu }-k\vec{A}.$$

### 4.1. Euler’s Equations

We will now demonstrate that a velocity field of the form in Equation (88), where the functions α, β, and ν satisfy the corresponding Equations (76), (82) and (77), necessarily obeys Euler’s equations. To achieve this, we first compute the material derivative of $\lambda \vec{v}$:

(91) $$\frac{d(\lambda \vec{v})}{dt}=\frac{d\vec{\nabla }\nu }{dt}+\frac{d\alpha }{dt}\vec{\nabla }\beta +\alpha \frac{d\vec{\nabla }\beta }{dt}+\frac{d\sigma }{dt}\vec{\nabla }s+\sigma \frac{d\vec{\nabla }s}{dt}-k\frac{d\vec{A}}{dt}$$

Without much effort, we derive

(92) $$\begin{array}{ccc} \frac{d\vec{\nabla }\nu }{dt} & = & \vec{\nabla }\frac{d\nu }{dt}-\vec{\nabla }v_{n}\frac{\partial \nu }{\partial x_{n}}=\vec{\nabla }\left(-\frac{c^{2}+w_{0}}{\gamma }+k\vec{A}\cdot \vec{v}-k\phi \right)-\vec{\nabla }v_{n}\frac{\partial \nu }{\partial x_{n}}\\ \frac{d\vec{\nabla }\beta }{dt} & = & \vec{\nabla }\frac{d\beta }{dt}-\vec{\nabla }v_{n}\frac{\partial \beta }{\partial x_{n}}=-\vec{\nabla }v_{n}\frac{\partial \beta }{\partial x_{n}}\\ \frac{d\vec{\nabla }s}{dt} & = & \vec{\nabla }\frac{ds}{dt}-\vec{\nabla }v_{n}\frac{\partial s}{\partial x_{n}}=-\vec{\nabla }v_{n}\frac{\partial s}{\partial x_{n}} \end{array}$$

in which we use the Cartesian coordinates $x_{n}$ and the summation convention. Inserting Equation (92) into Equation (91), it follows that

(93) $$\begin{array}{ccc} \frac{d(\lambda \vec{v})}{dt} & = & -\vec{\nabla }v_{n}\left(\frac{\partial \nu }{\partial x_{n}}+\alpha \frac{\partial \beta }{\partial x_{n}}+\sigma \frac{\partial s}{\partial x_{n}}\right)+\vec{\nabla }\left(-\frac{c^{2}+w_{0}}{\gamma }+k\vec{A}\cdot \vec{v}-k\phi \right)\\ & - & k\frac{d\vec{A}}{dt}+\frac{T_{0}}{\gamma }\vec{\nabla }s\\ & = & -\vec{\nabla }v_{n}\left(\lambda v_{n}+kA_{n}\right)+\vec{\nabla }\left(-\frac{c^{2}+w_{0}}{\gamma }+k\vec{A}\cdot \vec{v}-k\phi \right)\\ & - & k\partial_{t}\vec{A}-k\left(\vec{v}\cdot \vec{\nabla }\right)\vec{A}+\frac{T_{0}}{\gamma }\vec{\nabla }s\\ & = & -\frac{1}{\gamma }\vec{\nabla }w_{0}+k\vec{E}+k\left(v_{n}\vec{\nabla }A_{n}-v_{n}\partial_{n}\vec{A}\right)+\frac{T_{0}}{\gamma }\vec{\nabla }s, \end{array}$$

and we use Equation (8) of [25] as the definition of the electric field. Moreover, via Equation (7) of [25], the following equation is derived:

(94) $${\left(v_{n}\vec{\nabla }A_{n}-v_{n}\partial_{n}\vec{A}\right)}_{l}=v_{n}\left(\partial_{l}A_{n}-\partial_{n}A_{l}\right)=\epsilon_{lnj}v_{n}B_{j}={\left(\vec{v}\times \vec{B}\right)}_{l},$$

The Euler equation of a charged relativistic fluid is thus obtained,

(95) $$\frac{d(\lambda \vec{v})}{dt}=-\frac{1}{\gamma }\vec{\nabla }w_{0}+\frac{T_{0}}{\gamma }\vec{\nabla }s+k\left[\vec{v}\times \vec{B}+\vec{E}\right]=-\frac{1}{\rho }\vec{\nabla }P_{0}+k\left[\vec{v}\times \vec{B}+\vec{E}\right],$$

in which we have used Equation (19). Equation (95) is the same as Equation (51). We have thus proven that the Euler equations can be derived from the action principle (74). To summarize, all equations of relativistic charged fluid dynamics can be derived from the action principle (74).

### 4.2. Simplified Action

One might argue that the previous formulation complicates relativistic fluid dynamics by introducing four additional scalar fields, α, β, ν, and σ, beyond the physical variables $\vec{v}$, ρ, and *s*. However, this is only a superficial complication. We will show that the expression in Equation (71), written in a pedagogical form, can be simplified. To this end, we define a four-dimensional non-barotropic Clebsch four-vector:

(96) $$\begin{array}{ccc} v_{C}^{\mu } & \equiv & \alpha \partial^{\mu }\beta +\sigma \partial^{\mu }s+\partial^{\mu }\nu =\left(\frac{1}{c}\left(\alpha \partial_{t}\beta +\sigma \partial_{t}s+\partial_{t}\nu \right),\alpha \vec{\nabla }\beta +\sigma \vec{\nabla }s+\vec{\nabla }\nu \right)\\ & = & (\frac{1}{c}\left(\alpha \partial_{t}\beta +\sigma \partial_{t}s+\partial_{t}\nu \right),{\vec{v}}_{C}). \end{array}$$

In addition, we define a non-barotropic electromagnetic Clebsch four-vector:

(97) $$v_{E}^{\mu }\equiv v_{C}^{\mu }+kA^{\mu }=\left(v_{E}^{0},{\vec{v}}_{E}\right)=\left(\frac{1}{c}\left(\alpha \partial_{t}\beta +\sigma \partial_{t}s+\partial_{t}\nu +k\phi \right),{\vec{v}}_{C}-k\vec{A}\right).$$

Equations (76), (83) and (88) lead to the result

(98) $$v_{\mu }=-\frac{v_{E\mu }}{\lambda }\Rightarrow \;\vec{v}=\frac{{\vec{v}}_{E}}{\lambda },\;\lambda =-\frac{1}{c^{2}}\left(\alpha \partial_{t}\beta +\sigma \partial_{t}s+\partial_{t}\nu +k\phi \right).$$

The low-velocity (classical) limit of the above equations is

(99) $$\vec{v}≃{\vec{v}}_{E},\;\lambda ≃1.$$

$\vec{v}$ can now be eliminated from Equation (74), which (up to surface terms) takes the compact form

(100) $$\hat{L}\left[\rho_{0},s,\alpha ,\beta ,\nu ,\sigma \right]=\rho_{0}\left[c\sqrt{v_{E\mu }v_{E}^{\mu }}-\varepsilon_{0}-c^{2}\right]$$

This Lagrangian density produces the six Equations (76)–(78) and (82). Once these equations are solved, the scalar fields α, β, *s*, σ, and ν can be substituted into Equation (88) to determine $\vec{v}$. Consequently, the general problem of relativistic, charged, non-barotropic fluid dynamics is reformulated: instead of directly solving the Euler and continuity equations, one solves an equivalent set derived from the Lagrangian density $\hat{L}$. The classical limit of Equation (100) can then be found as follows. First, we observe that

(101) $$\sqrt{v_{E\mu }v_{E}^{\mu }}=|v_{E0}|\sqrt{1-\frac{{\vec{v}}_{E}^{2}}{v_{E0}^{2}}}=|v_{E0}|\sqrt{1-\frac{{\vec{v}}^{2}}{c^{2}}}=\frac{|v_{E0}|}{\gamma }=\frac{c|\lambda |}{\gamma }=c\left|\bar{\lambda }\right|$$

using Equation (98). According to the same equation, $v_{E0}<0$ as λ>0, and we obtain

(102) $$|v_{E0}|=-v_{E0}=-\frac{1}{c}\left(\alpha \partial_{t}\beta +\sigma \partial_{t}s+\partial_{t}\nu +k\phi \right).$$

Equations (101) and (102) are combined to derive

(103) $$c\sqrt{v_{E\mu }v_{E}^{\mu }}=-\frac{1}{\gamma }\left(\alpha \partial_{t}\beta +\sigma \partial_{t}s+\partial_{t}\nu +k\phi \right),$$

which can be expressed in terms of $\bar{\nu }$ (Equation (89)) in the following form:

(104) $$c\sqrt{v_{E\mu }v_{E}^{\mu }}=-\frac{1}{\gamma }\left(\alpha \partial_{t}\beta +\sigma \partial_{t}s+\partial_{t}\bar{\nu }+k\phi -c^{2}\right)$$

We can now formulate Equation (100) as follows:

(105) $$\begin{array}{ccc} \hat{L} & = & \rho_{0}\left[-\frac{1}{\gamma }\left(\alpha \partial_{t}\beta +\sigma \partial_{t}s+\partial_{t}\bar{\nu }+k\phi -c^{2}\right)-\varepsilon_{0}-c^{2}\right]\\ & = & -\frac{\rho_{0}}{\gamma }\left[\alpha \partial_{t}\beta +\sigma \partial_{t}s+\partial_{t}\bar{\nu }+k\phi +\gamma \varepsilon_{0}+\left(\gamma -1\right)c^{2}\right]. \end{array}$$

The classical limit is straightforward: $\frac{v}{c}\to 0,\gamma \to 1$. Note, however, that the final term should be handled with caution:

(106) $$\left(\gamma -1\right)c^{2}=\left(\frac{\gamma -1}{{\left(\frac{v}{c}\right)}^{2}}\right)v^{2}≃\frac{1}{2}v^{2}≃\frac{1}{2}v_{E}^{2}.$$

Using Equation (106), it follows that

(107) $$\hat{L}≃-\rho_{0}\left[\frac{\partial \bar{\nu }}{\partial t}+\alpha \frac{\partial \beta }{\partial t}+\sigma \frac{\partial s}{\partial t}+\varepsilon \left(\rho_{0},s_{0}\right)+k\phi +\frac{1}{2}{\left(\vec{\nabla }\bar{\nu }+\alpha \vec{\nabla }\beta +\sigma \vec{\nabla }s-k\vec{A}\right)}^{2}\right]$$

However, from Equation (24), it follows that

(108) $$\varepsilon_{0}=\frac{dE_{in\;\vec{\alpha }0}}{dM_{\vec{\alpha }}}=\gamma \frac{dE_{in\;\vec{\alpha }}}{dM_{\vec{\alpha }}}=\gamma \varepsilon .$$

We conclude that, in the (low-velocity) classical limit, there is no difference between ρ and $\rho_{0}$, and $\varepsilon_{0}$ and ε, since they differ by a term of order ${\left(\frac{v}{c}\right)}^{2}$. We thus obtain

(109) $$\hat{L}≃-\rho \left[\frac{\partial \bar{\nu }}{\partial t}+\alpha \frac{\partial \beta }{\partial t}+\sigma \frac{\partial s}{\partial t}+\varepsilon \left(\rho ,s\right)+k\phi +\frac{1}{2}{\left(\vec{\nabla }\bar{\nu }+\alpha \vec{\nabla }\beta +\sigma \vec{\nabla }s-k\vec{A}\right)}^{2}\right]$$

## 5. Energy–Momentum Tensor

We shall now calculate the canonical energy–momentum tensor of the Lagrangian density given in Equation (100); this is achieved through the definition given in Equation (60). However, as $\hat{L}$ is dependent only on the derivatives $\partial^{\mu }\beta ,\partial^{\mu }s,\partial^{\mu }\nu$, we obtain

(110) $$T_{E\mu \nu }=\partial_{\mu }\beta \frac{\partial \hat{L}}{\partial (\partial^{\nu }\beta )}+\partial_{\mu }s\frac{\partial \hat{L}}{\partial (\partial^{\nu }s)}+\partial_{\mu }\nu \frac{\partial \hat{L}}{\partial (\partial^{\nu }\nu )}-\eta_{\mu \nu }\hat{L}.$$

Taking into account Equation (101), it is easy to see that

(111) $$\frac{\partial \hat{L}}{\partial (\partial^{\nu }\beta )}=\frac{\rho \alpha }{\lambda }v_{E\nu },\;\frac{\partial \hat{L}}{\partial (\partial^{\nu }s)}=\frac{\rho \sigma }{\lambda }v_{E\nu },\;\frac{\partial \hat{L}}{\partial (\partial^{\nu }\nu )}=\frac{\rho }{\lambda }v_{E\nu }.$$

Plugging Equation (111) into Equation (110), we have

(112) $$T_{E\mu \nu }=\frac{\rho }{\lambda }\left(\alpha \partial_{\mu }\beta +\sigma \partial_{\mu }s+\partial_{\mu }\nu \right)v_{E\nu }-\eta_{\mu \nu }\hat{L}=\frac{\rho }{\lambda }v_{C\mu }v_{E\nu }-\eta_{\mu \nu }\hat{L},$$

in which we have used the definition given in Equation (96). The above formula can be expressed in terms of more physical velocity fields using Equations (97) and (98) as

(113) $$T_{E\mu \nu }=\rho \left(\lambda v_{\mu }+kA_{\mu }\right)v_{\nu }-\eta_{\mu \nu }\hat{L}.$$

By using Equation (101), we notice that Equation (100) can be formulated as

(114) $$\hat{L}=\rho_{0}\left[c^{2}\bar{\lambda }-\varepsilon_{0}-c^{2}\right].$$

Taking into account Equation (41), we see that $\hat{L}$ is numerically equal to the pressure at the rest frame,

(115) $$\hat{L}=\rho_{0}\left[c^{2}\left(1+\frac{w_{0}}{c^{2}}\right)-\varepsilon_{0}-c^{2}\right]=\rho_{0}\left[w_{0}-\varepsilon_{0}\right]=P_{0},$$

in which, for the last equality sign, we have used Equation (17). Thus,

(116) $$T_{E\mu \nu }=\rho \left(\lambda v_{\mu }+kA_{\mu }\right)v_{\nu }-\eta_{\mu \nu }P_{0}.$$

It is interesting to compare this expression to the one suggested by Weinberg [15]; to do this, we look at uncharged fluids for which k=0. Using Equations (41) and (73), we derive

(117) $$\rho \lambda v_{\mu }v_{\nu }=\rho \lambda \frac{u_{\mu }u_{\nu }}{\gamma^{2}}=\rho_{0}\bar{\lambda }u_{\mu }u_{\nu }=\rho_{0}\left(1+\frac{w_{0}}{c^{2}}\right)u_{\mu }u_{\nu }=\rho_{0}\left(1+\frac{\varepsilon_{0}+\frac{P_{0}}{\rho_{0}}}{c^{2}}\right)u_{\mu }u_{\nu }$$

Thus,

(118) $$\rho \lambda v_{\mu }v_{\nu }=\frac{1}{c^{2}}\left(\rho_{0}c^{2}+\rho_{0}\varepsilon_{0}+P_{0}\right)u_{\mu }u_{\nu }=\frac{1}{c^{2}}\left(e_{0}+P_{0}\right)u_{\mu }u_{\nu }$$

in which we have used the definition given in Equation (54). Thus, we may rewrite the energy–momentum tensor of an uncharged flow in the form

(119) $$T_{E\mu \nu }=\frac{1}{c^{2}}\left(e_{0}+P_{0}\right)u_{\mu }u_{\nu }-\eta_{\mu \nu }P_{0}.$$

This should be compared to the Weinberg [15] form

(120) $$T_{W\mu \nu }=\left(\rho_{W}+P_{W}\right)u_{W\mu }u_{W\nu }+\eta_{W\mu \nu }P_{W}.$$

in which we need to take into account that Weinberg does not use practical MKS units as we do but natural units in which c=1; thus, $u_{W\mu }=u_{\mu }$. The metric that Weinberg uses is also in contrast to our own; hence, $\eta_{W\mu \nu }=-\eta_{\mu \nu }$. It also follows that Weinberg’s pressure is the pressure at the rest frame $P_{w}=P_{0}$ and that Weinberg’s proper energy density is the total energy density in the rest frame: $\rho_{w}=e_{0}$.

## 6. Conclusions

Current cosmological models—in particular, ΛCDM—postulate an unconfirmed component (the Λ in ΛCDM) that is denoted as “dark energy”. This is needed to fit the accelerating expansion of the universe as obtained through distant supernovae observations, the CMB spectrum, and the large-scale structure that developed through baryonic acoustic oscillations in primordial matter. The energy–momentum tensor obtained in Equation (116) allows us two alternative routes. One is to assume that the primordial fluid was not electrically neutral and thus one must take into account the electromagnetic contributions to the energy–momentum tensor. The other one is to take into account a non-barotropic fluid in which the quantity $\rho_{0}\varepsilon_{0}$ is not merely a simple multiplication of $P_{0}$, which may be either 3 or $\frac{3}{2}$ depending on wether the fluid is ultra-relativistic or non-relativistic. Rather, we use a more realistic form in which $\rho_{0}\varepsilon_{0}$ is dependent on two thermodynamical quantities, which can be either the density, specific entropy, pressure, or temperature, all defined in the fluid element’s rest frame. Of course, more detailed analysis is needed, which will hopefully be published in future papers.

## Data Availability

No new data were created or analyzed in this study. Data sharing is not applicable to this article.
